# ^18^F-FDG PET/CT radiomics nomogram for predicting occult lymph node metastasis of non-small cell lung cancer

**DOI:** 10.3389/fonc.2022.974934

**Published:** 2022-09-28

**Authors:** Jianyi Qiao, Xin Zhang, Ming Du, Pengyuan Wang, Jun Xin

**Affiliations:** ^1^ Department of Radiology, Shengjing Hospital of China Medical University, Shenyang, China; ^2^ Department of Nuclear Medicine, Shengjing Hospital of China Medical University, Shenyang, China

**Keywords:** nomogram, non-small cell lung cancer, occult lymph node metastasis, radiomics, PET/CT

## Abstract

**Purpose:**

To investigate the ability of a PET/CT-based radiomics nomogram to predict occult lymph node metastasis in patients with clinical stage N0 non-small cell lung cancer (NSCLC).

**Materials and methods:**

This retrospective study included 228 patients with surgically confirmed NSCLC (training set, 159 patients; testing set, 69 patients). ITKsnap3.8.0 was used for image(CT and PET images) segmentation, AK version 3.2.0 was used for radiomics feature extraction, and Python3.7.0 was used for radiomics feature screening. A radiomics model for predicting occult lymph node metastasis was established using a logistic regression algorithm. A nomogram was constructed by combining radiomics scores with selected clinical predictors. Receiver operating characteristic (ROC) curves were used to verify the performance of the radiomics model and nomogram in the training and testing sets.

**Results:**

The radiomics nomogram comprising six selected features achieved good prediction efficiency, including radiomics characteristics and tumor location information (central or peripheral), which demonstrated good calibration and discrimination ability in the training (area under the ROC curve [AUC] = 0.884, 95% confidence interval [CI]: 0.826-0.941) and testing (AUC = 0.881, 95% CI: 0.8031-0.959) sets. Clinical decision curves demonstrated that the nomogram was clinically useful.

**Conclusion:**

The PET/CT-based radiomics nomogram is a noninvasive tool for predicting occult lymph node metastasis in NSCLC.

## Introduction

Lung cancer is one of the most common cancers and is the leading cause of cancer-related deaths worldwide, with two million new cases and 1.79 million deaths occurring per year (1, 2). Non-small cell lung cancer (NSCLC) accounts for approximately 85% of all lung cancer cases (3). Mediastinal staging is critical for patients with NSCLC without systemic metastases as it provides accurate information on the extent of disease, guides treatment options, and determines patient prognosis (4). Mediastinoscopy and endobronchial ultrasound-guided transbronchial needle aspiration (EBUS-TBNA) is the current gold standard for preoperative lymph node staging but is not routinely recommended because of its invasive nature (5–8). Among preoperative noninvasive diagnostic methods, positron emission tomography (PET)/computed tomography (CT) is used as an imaging modality that provides information on anatomy and glucose metabolism. This approach has emerged as a key noninvasive lung cancer staging method (9, 10) and exhibits better performance for lung cancer lymph node staging (estimated sensitivity of 77% and specificity of 86%) compared to CT (55% and 81%, respectively) (11). Nevertheless, lymph node micrometastases cannot be detected using PET/CT, referred to herein as occult lymph node metastasis (OLM). OLM means that there are no suspicious lymph nodes in hilar and mediastinum by PET/CT before operation, but the pathological results after operation confirm that there are lymph node metastases. In patients with negative PET/CT lymph node uptake, the incidence of OLM is 14.3–23.1% (12, 13). Lung resection with systematic nodal dissection is the main treatment option for stage I and II NSCLC,and total lobectomy is considered the preferred lung resection method (14, 15). However, some studies have reported that there is no significant difference in the prognosis of sublobectomy and lobectomy in patients with early stage lung cancer (stage I) without lymph node metastasis. Sublobar resection can reduce the incidence of complications and preserve lung function (16–18). With regard to prognosis, preoperative lymph node status is related to patient prognosis and survival rate. In patients clinically diagnosed with N0 stage, survival rate is significantly lower for patients with NSCLC with OLM than for those without OLM (19). As such, the accurate evaluation of OLMs in lung cancer will facilitate the assessment of patient prognosis and help to guide treatment.

The concept of radiomics was first proposed by the Dutch scholar Iambin (20). Using high-throughput calculations, it is now possible to rapidly extract a large number of quantitative features from radiological images. Radiomics refers to the process of converting digital medical images into high-dimensional data that can be mined (21). Several reports have highlighted the utility of radiomics for the diagnosis, staging, prognosis, and efficacy analysis of lung cancer (22–25). At present, there is a paucity of studies on OLM of NSCLC (26–29). Several reports have demonstrated that radiomics models based on CT and enhanced CT have predictive value for OLM (26–28). A recent study used the texture parameters of PET images combined with metabolic parameters (MTV) and serological data (CEA) to construct a radiomics nomogram, which achieved good prediction results (29). Most radiomics studies on OLM related to NSCLC have been based on CT or PET images, and there is a lack of literature on texture information of PET and CT images simultaneously. In this study, we aimed to construct a radiomics nomogram to predict OLM in patients with clinical stage N0 NSCLC by synthesizing CT and PET texture and harnessing clinical data of patients.

## Materials and methods

### Patients

Our hospital ethics committee approved this retrospective study. All patients who underwent preoperative whole-body PET/CT imaging between March 2012 and July 2021 were searched in the institutional database. We included patients who met the following criteria: (1) underwent surgical resection and systemic lymphadenectomy; (2) the tumor was confirmed to be adenocarcinoma(ADC) or squamous cell carcinoma(SCC) by histopathological examination postoperatively; (3) PET/CT examination was performed within 1 month preoperatively; and (4) the primary lesion is single (5)the lesion was diagnosed as clinical stage N0 (cN0), short axis of all lymph nodes was < 10 mm, SUVmax of mediastinal lesions was < 2.5, and there was no distant metastasis (M0). Patients who met any of the following criteria were excluded: (1) the lesion exhibited ground-glass density on CT images; (2) lesion boundaries were unclear owing to respiratory artifacts or inflammation around the tumor; and (3) patients who received chemotherapy or radiation before undergoing PET/CT. A total of 228 patients were identified, comprising 85 OLM and 143 non-OLM cases. To ensure a balanced proportion of OLM and non-OLM cases in each dataset, we used a stratified sampling method (stratified by label) to divide the data into a training set (n = 159) and testing set (n = 69) at a ratio of 7:3 using Python 3.7.0. Clinical features of the patients were recorded. Clinical data including age, sex, smoking history, and serological examination (CEA) for each patient were obtained from medical records. CT features (tumor long and short diameters, tumor location, and lung lobe in which the tumor was located) were recorded. The classification criteria for tumor location were as follows: tumor center located within one-third of the lung parenchyma was defined as central lung cancer, otherwise the tumor was defined as peripheral lung cancer. The maximum standardized uptake value (SUVmax), mean standardized uptake value (SUVmean), and metabolic tumor volume (MTV) of primary lesions were automatically measured on GE Advanced Workstation (AW, V4.5). Total lesion glycolysis (TLG) was calculated using the common formula (TLG = SUVmean × MTV) (30).

### PET/CT image acquisition

All patients underwent the same PET/CT examination with the same acquisition parameters. Patients fasted for more than 6 hours before the examination to control blood glucose levels below 11.10 mmol/L. All patients received GEDiscoveryElitePET/CT scans from the skull base to femur 1\2 after intravenous injection of ^18^F-FDG at 3.70-5.55 Mbq/Kg and 1 hour after drug absorption. After selecting the examination range, spiral CT scan was performed. The tube voltage was 140 kV, automatic tube current was 180-240 mA, and layer thickness was 3.75 mm. PET images were then collected from 6-7 beds at a rate of 1.5 minutes/bed. A 3D-ordered subset expectation maximization algorithm was used to reconstruct PET images after attenuation correction.

### Image preprocessing, tumor segmentation, and feature extraction

**Figure 1:**
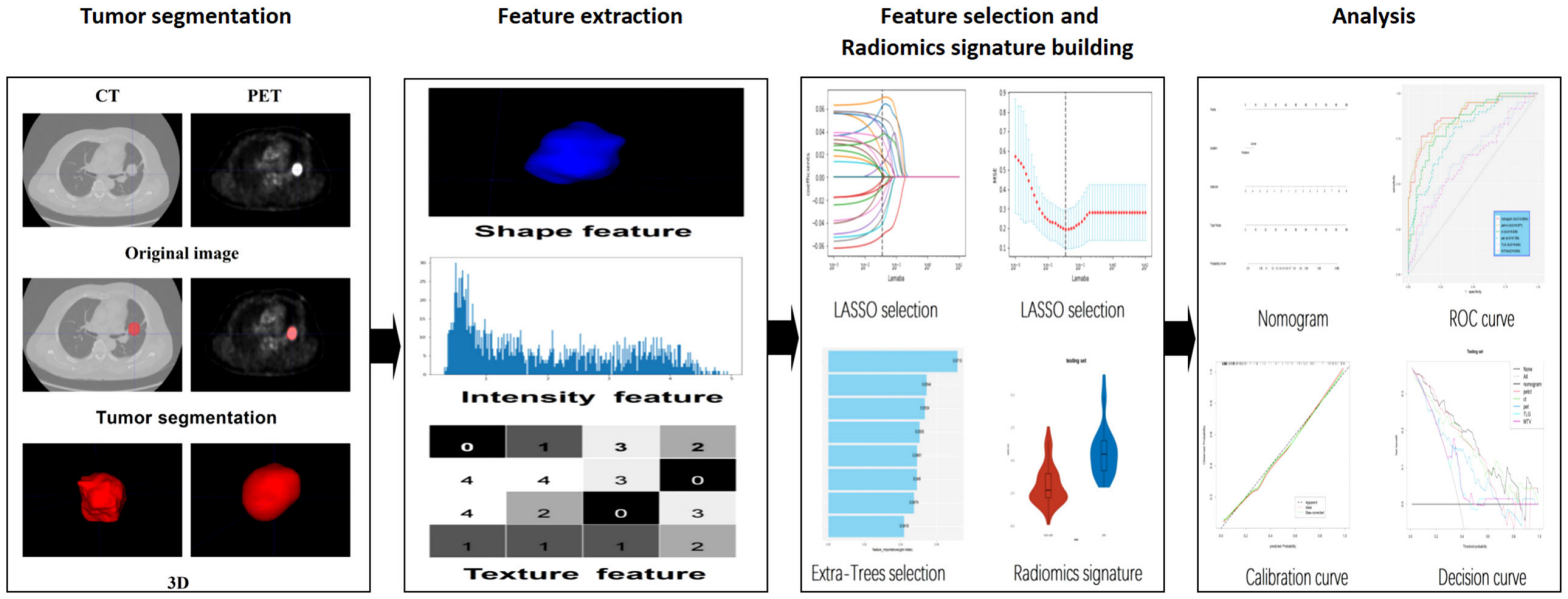
Workflow implemented in this study.

**Figure 1** depicts the study flowchart. Image preprocessing was completed using the artificial intelligence Kit (A.K, version 3.2.0, GE Healthcare). A linear interpolation algorithm was used to resample the thickness of CT images to 1 mm. Gaussian filtering was used to process CT images. Tumor segmentation was then performed by a nuclear medicine physician with 6 years of radiology experience using ITKsnap3.8.0. On preprocessed images of all patients, semi-automatic (adaptive brush tool in ITKsnap3.8.0) and manual methods were used to draw the region of interest (ROI). Radiologists were informed about the location where the tumor was confirmed but were blinded to other clinical information and pathological outcomes. The segmentation results were then verified by a senior radiologist with 20 years of experience. A total of 1316 radiomics features were extracted from the ROI of CT and PET images using AK software, including first-order, shape, gray level cooccurrence matrix (GLCM), gray level dependence matrix (GLDM), gray level run-length matrix (GLRLM), gray level size zone matrix (GLSZM), and neighboring gray tone difference matrix (NGTDM) features. To obtain more effective features, we made the following three changes to the original images: local binary pattern, Gaussian Laplacian filtering, and wavelet. Two sets of radiomics features were combined (1316 × 2) as radiomics features for PET/CT.

### Feature selection and establishment of radiomics signature

**Figure 2:**
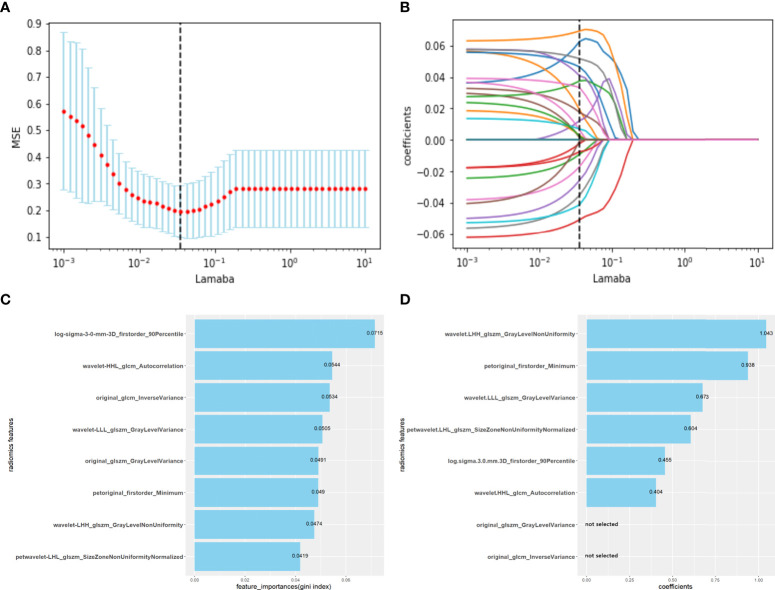
Radiomics features selected using the LASSO regression model, extremely randomized trees and logistic regression backward selection method. **(A)** The abscissa is the penalty coefficient λ, the ordinate is the mean squared error (MSE), and the vertical line is the λ corresponding to the lowest MSE, that is, the optimal penalty coefficient (λ=0.03556). **(B)** The ordinate is the coefficient of features. There are 23 non-zero coefficients when λ takes the optimum penalty coefficient. **(C)** Using extremely randomized trees, eight corresponding features with high importance were selected according to the Gini coefficient. **(D)** Using logistic regression backward selection method, two features were discarded, and six features were retained. Radiomics score (radscore) formula: radscore =Σ (radiomic features *coefficients) + intercept (-0.7135).

Z-scores were used to standardize the radiomics parameters of all patients. A t-test was used to analyze features that conformed to a normal distribution and had a homogeneous variance; otherwise, the Mann−Whitney U test was used. The 10-fold least absolute shrinkage and selection operator (LASSO) algorithm, extremely randomized trees (extra-tree), and logistic regression backward selection were used to select the most useful predictive radiomics features from the training set. The radiomics score (radscore) formula was generated using a linear combination of selected features weighted by their respective coefficients (**Figure 2**). The radscore of each patient was calculated using this formula to compare the radscores of OLM and non-OLM cases. The prediction efficiency of radiomics features was quantified using the area under the receiver operating characteristic (ROC) curve (AUC) in the training and testing sets.

### Selection of clinical predictors and construction of radiomic nomogram

For the CEA data with missing values (six in the training set and four in the testing set), the missing values were replaced with the average value of CEA in the training set. Univariate and multivariate logistic regression was used to screen the independent predictors associated with the identification of NSCLC OLM from clinical characteristics and radscore. A radiomic nomogram was constructed based on a multivariate logistic regression model. The AUC was used to assess radiomic predictive efficacy, and the Delong test was used to compare AUC between groups. Calibration curves and the Hosmer–Lemeshow (HL) test were used to evaluate calibration performance. The clinical usefulness of the radiomics nomogram was evaluated using decision curve analysis (DCA).

### Statistical analysis

Statistical analyses were performed using R software 4.1.2 and python 3.7.0 software. Clinical measurement data conforming to a normal distribution are expressed as mean ± Standard Deviation(SD), whereas measurement data not conforming to a normal distribution are expressed as quartiles and medians. Two groups were compared using an independent-samples t-test or Mann−Whitney U-test. The constituent ratio of counting data was tested using a four-grid table chi-squared test and two-tailed test. Statistical significance was set at *P* < 0.05. The packages of R and python used in the article are shown in the **Supplementary Material**

## Results

### Clinical characteristics

The clinical characteristics of patients in the training and testing sets are summarized in **Table 1**. In total, 159 and 69 patients were assigned to the training and testing sets, respectively. There were no significant differences between the training and testing sets, with the exception of age.

**Table 1:** Clinical Characteristics of Patients in the Training and Testing Sets.

		Training set			Testing set						P-value
Characteristics		NON-OLM	OLM	P-value	NON-OLM	OLM	P-value			
**n**		100	59		43	26		
**Sex (%)**	Female	50 (50.0)	29 (49.2)	1	21 (48.8)	15 (57.7)	0.642				0.841
	Male	50 (50.0)	30 (50.8)		22 (51.2)	11 (42.3)		
**Age (median [IQR])**		61.50[55.00,67.00]	59.00 [53.00, 64.50]	0.195	64.00[57.00,68.00]	63.00[60.25,64.75]	0.911				0.036
**Pathological type (%)**	Adenocarcinoma	74 (74.0)	47 (79.7)	0.538	35 (81.4)	19 (73.1)	0.61				0.854
	Squamous cell carcinoma	26 (26.0)	12 (20.3)		8 (18.6)	7 (26.9)		
**Lobe (%)**	LLL	22 (22.0)	10 (16.9)	0.487	8 (18.6)	3 (11.5)	0.008				0.799
	LUL	20 (20.0)	18 (30.5)		8 (18.6)	9 (34.6)		
	RLL	21 (21.0)	14 (23.7)		7 (16.3)	9 (34.6)		
	RML	7 (7.0)	2 (3.4)		0 (0.0)	2 (7.7)		
	RUL	30 (30.0)	15 (25.4)		20 (46.5)	3 (11.5)		
**Location (%)**	Central	9 (9.0)	23 (39.0)	<0.001	1 (2.3)	10 (38.5)	<0.001				0.577
	Peripheral	91 (91.0)	36 (61.0)		42 (97.7)	16 (61.5)		
**Tumor long diameter (median [IQR])**		2.70 [2.10,3.40]	3.20 [2.30,4.40]	0.005	2.50 [1.80, 2.90]	3.20 [2.90, 3.68]	<0.001				0.27
**Tumor short diameter (median [IQR])**		2.10 [1.70,2.70]	2.80 [1.90,3.40]	0.001	1.90 [1.50, 2.50]	2.50 [2.10, 3.10]	0.005				0.425
**CEA (median [IQR])**		3.08 [1.81,5.61]	4.00 [2.27,7.32]	0.039	2.69 [1.91, 6.20]	3.08 [2.09, 6.28]	0.752				0.533
**Smoking (%)**	No	56 (56.0)	36 (61.0)	0.651	22 (51.2)	17 (65.4)	0.366				0.966
	Yes	44 (44.0)	23 (39.0)		21 (48.8)	9 (34.6)		
**SUVmax (median [IQR])**		9.14[6.14,12.96]	9.87 [7.64, 13.73]	0.212	8.58 [6.38, 11.85]	10.64 [7.98, 18.34]	0.022				0.771
**SUVmean (median [IQR])**		5.40 [3.53, 7.90]	6.25[4.60,8.75]	0.213	5.18 [3.89, 7.28]	6.68 [4.80, 10.83]	0.022				0.752
**MTV (median [IQR])**		5.15 [2.68, 8.71]	8.21[3.58,19.88]	0.005	4.69 [2.71, 6.52]	9.15 [4.94, 12.80]	0.002				0.461
**TLG (median [IQR])**		26.14[12.48,48.68]	47.55[19.63,103.28]	0.002	17.39 [8.41, 42.37]	58.47 [25.66, 102.04]	<0.001				0.677

P-value of the last column show differences of variables in training set and testing set.

IQR, interquartile range; LLL, left lower lobe; LUL, left upper lobe; RLL, right lower lobe; RML, right middle lobe; RUL, right upper lobe.

### MTV and TLG evaluation of OLM

Reports suggest that the MTV and TLG are good independent predictors of OLM (31, 32). We used MTV and TLG to predict OLM and evaluated prediction efficacy using AUCs. The AUCs of the training set were 0.634 (CI: 0.542-0.726) and 0.6439 (CI: 0.554-0.734), while the AUCs of the testing set were 0.724 (CI: 0.595-0.854) and 0.762 (CI: 0.645-0.879) (**Table 3**, and **Figures 5A, B**).

### Construction of radiomics signature

**Figure 3:**
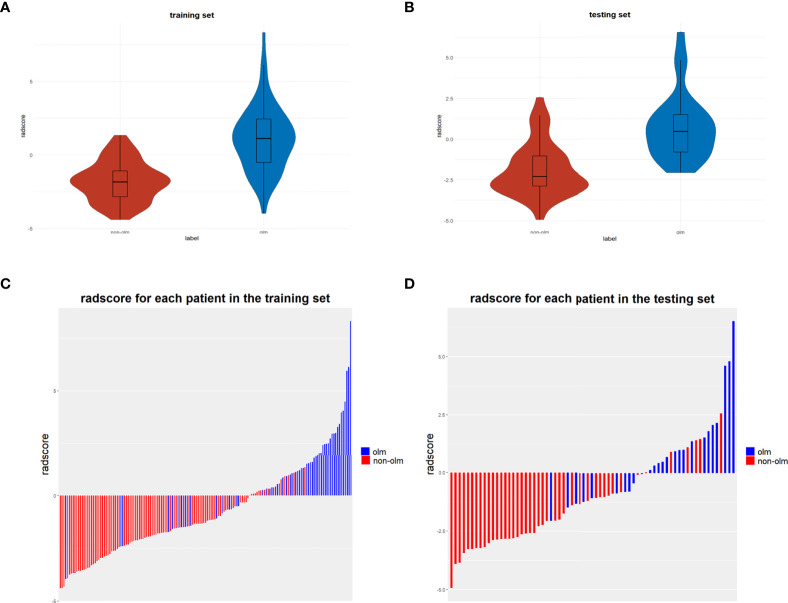
**(A, B)** Violin distributions of the radscore of benign and malignant nodes in the training and testing sets. In each violin plot, the middle horizontal line and upper and lower edges of the rectangle represent the median and upper and lower quartiles (Q1, Q3), respectively, and the two ends of the vertical line are the extreme value points. **(C, D)** bar graphs show the rad-scores of patients in the training and testing sets.

**Figure 4:**
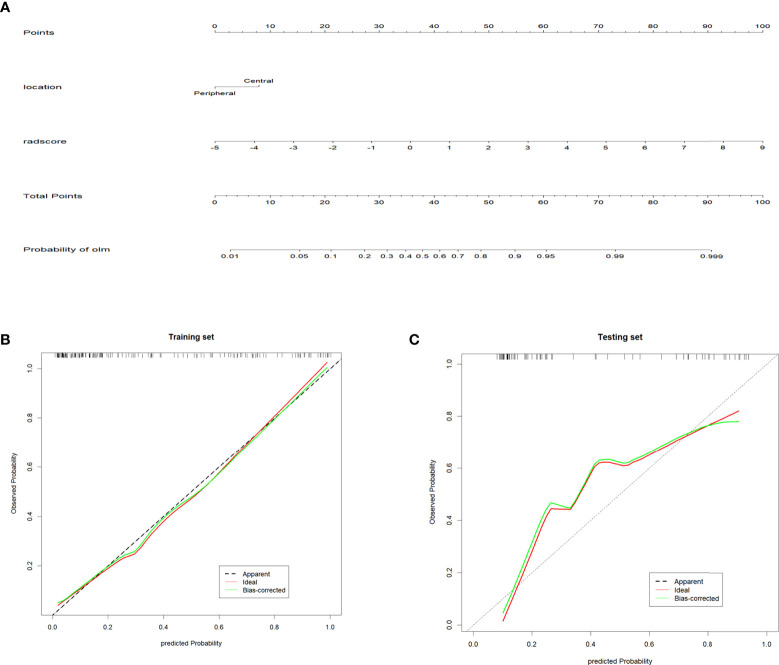
Radiomics nomogram and Calibration curves **(A)** Radiomics nomogram for predicting occult lymph node metastasis of non-small cell lung cancer. **(B, C)** Calibration curve of the radiomics nomogram in the training and testing sets. The diagonal dotted line represents the ideal prediction. The solid line represents the performance of the nomogram. The closer to the diagonal dotted line, the better the prediction effect.

**Figure 5:**
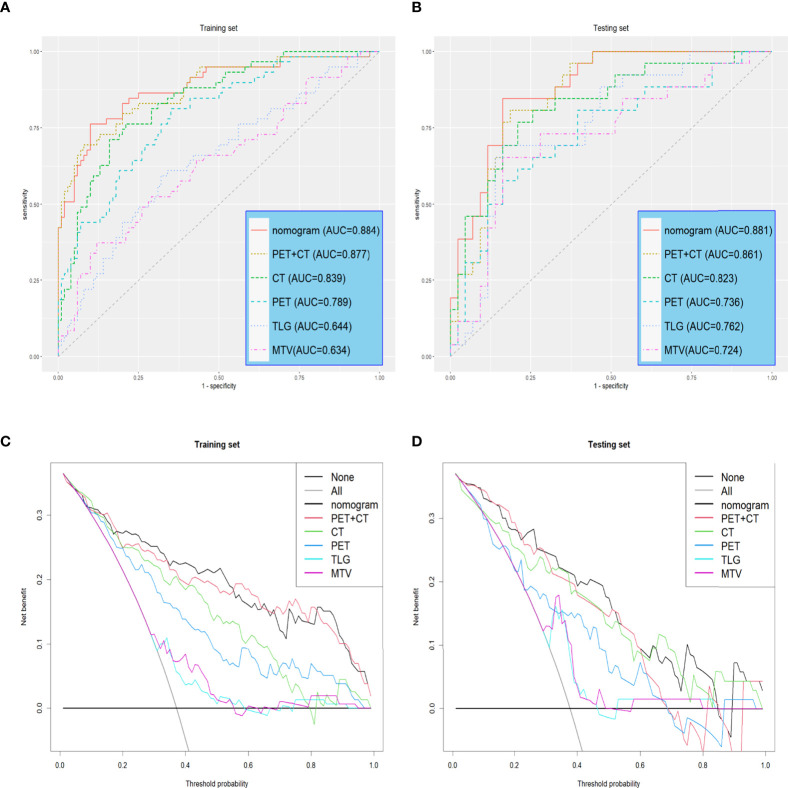
Receiver operating characteristic (ROC) curves and Decision curves **(A, B)** Receiver operating characteristic (ROC) curves of all models in the training and testing sets. **(C, D)** Decision curves of all models in training and testing sets. The gray line indicates that all patients are considered OLM cases. The black line indicates that all patients are considered non-OLM cases.

A total of 1316 radiomic features were extracted from the ROI for each CT and PET image and were combined as PET+CT radiomic features. A t-test or U test, LASSO algorithm, and extra-tree were used to screen out eight OLM-related radiomic features. The prediction model was constructed using a binary logistic regression backward selection method. Finally, two features were excluded, leaving six radiomic features (**Figure 2**). The radscore for each case was calculated based on six radiomic features. Significant differences were observed in radscores (median [interquartile range]) between OLM cases and non-OLM cases (1.12 [-0.50, 2.44] and -1.83 [-2.83, - 1.09], respectively; *P* < 0.001, Mann−Whitney U test). This difference was confirmed in the testing set (0.46 [-0.80, 1.49] and -2.30 [-2.87, -1.03], respectively; *P* < 0.001) (**Figure 3**). The radiomics scores of CT and PET imaging were constructed in the same manner. The radscore of PET/CT radiomics will be used for the establishment of nomogram (**Figure 4**). Radscores were evaluated using ROC curves and AUCs (**Figure 5**). Predictive performance was higher for the radscore of PET/CT radiomics than for CT radiomics and PET radiomics alone.

### Construction of radiomics nomogram

Univariate logistic regression analysis revealed that radscore, tumor long diameter, tumor short diameter, tumor location, MTV, and TLG were correlated with OLM. Multiple logistic regression backward selection identified tumor location and radscore as independent predictors (**Table 2**). Therefore, we developed a nomogram that combined radscore and tumor location (**Figure 4**). The AUCs of the nomogram were 0.884 (CI: 0.826-0.941) and 0.881 (CI: 0.803-0.959) for the training and testing sets, respectively (**Table 3** and **Figures 5A, B**). These values were not significantly different to the AUCs of the PET/CT radiomics (Delong test, training set: *P* = 0.880; testing set: *P* = 0.728) but were significantly different to the AUCs of MTV (Delong test, training set:*P* < 0.001; testing set: *P* = 0.013) and TLG (Delong test, training set:*P* < 0.001; testing set: *P* = 0.032). Calibration curves (**Figures 4B, C**) and Hosmer–Lemeshow test results (training set: *P* = 0.820, testing set: *P* = 0.455) revealed that the predicted probability of the nomogram was consistent with the actual probability of OLM (33). The decision curves for the six models in the training and testing sets are presented in **Figures 5C, D**, respectively. On the whole, the net benefit of all models was higher than assuming that all patients had OLM, and the net benefit of the nomogram was higher than that of other models (34).

**Table 2:** Risk Factors for Patients with OLM.

	Univariate logistic regression		Multivariate logistic regression(backward selection)		Final nomogram	
	OR (95% CI)	P value	OR (95% CI)	P value	OR (95% CI)	P value
**Location**	6.460 (2.729, 15.294)	***<0.001***	3.43 (1.172,10.055)	***0.025***	2.843 (1.007,8.028)	***0.048***
**Tumor long diameter**	1.587 (1.203,2.904)	***0.001***	Not selected		Not selected	
**Tumor short diameter**	1.901 (1.331,2.717)	***<0.001***	Not selected		Not selected	
**MTV**	1.035 (1.008,1.063)	***0.010***	Not selected		Not selected	
**TLG**	1.003 (1.000,1.006)	***0.022***	0.997 (0.994, 1.000)	0.088	Not selected	
**Radscore**	2.718 (1.994,3,706)	***<0.001***	2.773 (1.970,3.903)	***<0.001***	2.543 (1.860,3.477)	***<0.001***
**Sex** **Age** **Pathological type** **Lobe LLL** ** LUL** ** RLL** ** RML** ** RUL** ** CEA** **Smoking** **SUVmax** **SUVmean**	0.967 (0.508, 1.840) 0.971 (0.932,1.011) 1.376 (0.634,2.989) 0.909 (0.344,2.401) 1.800 (0.740,4.377) 1.333 (0.533,3.337) 0.571 (0.106,3.095) ——— 1.045 (0.999,1.093) 1.230 (0.639,2.369) 1.039 (0.978,1.104) 1.057 (0.959,1.164)	0.918 0.152 0.420 0.847 0.195 0.539 0.516 0.498 0.056 0.536 0.216 0.263				

Lobe is an unordered multi categorical variable, which we convert into a dummy variable, and RUL is used as a reference.

Bold values represent p-values less than 0.05.

**Table 3:** Performance of the Predictive Model.

	Data set	AUC (95% CI)	Accuracy	Sensitivity	Specificity	PPV	NPV
**Nomogram**	Training set	0.884 [0.826-0.941]	0.843	0.763	0.89	0.804	0.864
	Testing set	0.881 [0.803-0.959]	0.797	0.654	0.884	0.773	0.809
**PET + CT**	Training set	0.877 [0.819-0.936]	0.818	0.729	0.870	0.768	0.845
	Testing set	0.861 [0.775-0.946]	0.768	0.615	0.860	0.727	0.787
**CT**	Training set	0.839 [0.776-0.901]	0.774	0.627	0.860	0.725	0.796
	Testing set	0.823 [0.719-0.927]	0.754	0.615	0.837	0.696	0.783
**PET**	Training set	0.789 [0.717-0.861]	0.717	0.525	0.83	0.646	0.748
	Testing set	0.736 [0.610-0.862]	0.681	0.346	0.884	0.643	0.691
**TLG**	Training set	0.644 [0.554-0.734]	0.648	0.593	0.680	0.522	0.739
	Testing set	0.762 [0.645-0.879]	0.681	0.692	0.674	0.562	0.784
**MTV**	Training set	0.634 [0.542-0.726]	0.373	0.880	0.647	0.704	0.373
	Testing set	0.724 [0.595-0.854]	0.231	0.907	0.600	0.661	0.231

PPV, positive predictive value; NPV, negative predictive value.

## Discussion

PET/CT is recommended for the diagnosis and staging of lung cancer and is more effective for diagnosing lymph node metastasis in lung cancer compared to traditional CT (11, 35). However, a subset of micrometastatic lymph nodes cannot be detected by PET/CT, referred to as OLM. Accurate prediction of lymph node micrometastasis can guide surgical treatment decision-making and is closely associated with patient prognosis. We propose a PET/CT based nomogram that combines radscore and location information of the tumor (central or peripheral). This nomogram successfully predicted OLM in patients with NSCLC. The predictive ability of this nomogram was higher than that of MTV, TLG, and three radiomics models (CT, PET, and PET + CT). As a noninvasive method, our model may assist in the diagnosis of lymph node metastasis of NSCLC and improve the positive detection rate on PET/CT imaging.

ADC and SCC are the most common subtypes of NSCLC, accounting for approximately 60% and 35–40% of NSCLCs, respectively (36). Large cell lung cancers and other types of NSCLCs account for less than 5% of cases. Large cell lung cancer is more malignant than ADC and SCC, and is more prone to early metastasis (37, 38). Therefore, when selecting patients for this study, we excluded other types of NSCLC, except ADC and SCC, to reduce potential confounding effects of different pathological types on results of the model. In addition, we exclude ground-glass density nodules because the hounsfield unit(CT) and SUV(PET) of ground glass nodules are relatively low, so it is difficult to draw the ROI on CT and PET images. Moreover, ground-glass density nodules are generally early lung cancer and rarely have lymph node metastasis.

Mediastinoscopy and EBUS-TBNA are the current gold standards for preoperative lymph node staging. However, these approaches are invasive, and false-negative results may occur. Reports suggest that the metabolic parameters of primary lesions of NSCLC can be used to predict OLM (31, 32, 39–41). Kim et al. reported that high SUVmax and MTV displays were associated with an increased risk of OLM. Park et al. argued that SUVmax and MTV were independent risk factors for OLM and that MTV was a better predictor (31). Shin et al. reported that TLFsur was the most effective factor for predicting OLM in cN0 lung adenocarcinoma (32). In our study, univariate logistic regression analysis identified MTV and TLG as independent predictors of OLM, which was consistent with previous results. However, SUVmax was not included. One possible explanation is that SUVmax only represents a single metabolic focus with the highest voxel value in the ROI and is susceptible to interference from noise and resolution (32, 42, 43). Therefore, SUVmax may not be the best choice to reflect the metabolic state of the tumor. MTV, TLG, and other indicators may reflect more metabolic information in some cases, as MTV and TLG only reflect the tumor generation status and lack information on tumor morphology, location, and clinical information. This may partly explain the lower effectiveness of MTV and TLG alone for predicting OLM compared to PET/CT radiomics models and nomograms.

To improve the detection rate of lymph node metastases by PET/CT and avoid invasive operations and complications, we constructed a radiomics nomogram using the 10-fold LASSO algorithm, extra- tree, and other methods to reduce the number of radiomic features. This approach, which is widely employed in the process of radiomic feature selection, is more suitable for the reduction of high-dimensional data and can reduce the multicollinearity between features (44, 45). An extremely randomized tree classifier expresses the importance of features by calculating the Gini index of each feature. Then, the corresponding number of variables with high importance is selected according to the needs. In this study, variables with a Gini index higher than the average value were selected (46). Finally, six variables were incorporated into the model, including two PET (one first-order and one GLCM feature) and four CT radiomics features (one first-order, one GLCM, and two GLSZM features). Radscore was incorporated into the nomogram, as well as tumor location (central or peripheral). Numerous studies have demonstrated that the risk of lymph node metastasis is significantly higher in central lung cancer than in peripheral lung cancer (47–49). Decaluwe reported that all five definitions of central lung cancer could predict lymph node metastasis in NSCLC (48). Moulla et al. reported that patients with NSCLC with large tumors and central tumor location required accurate preoperative and intraoperative evaluation of lymph node status because of the increased risk of lymph node metastasis. They recommended invasive mediastinal staging by EBUS-TBNA or television mediastinoscopy for patients with central tumors and mediastinal negativity (49). When tumor location was included as an independent risk factor in our nomogram, the model’s efficiency was improved (AUCs of the training and testing sets were 0.884 > 0.877 and 0.881 > 0.861, respectively).

Our study has several limitations. For example, this study was a single-center study and lacked external validation to confirm the reliability of the model. The data were unbalanced, with more negative than positive data. In addition, this model is applicable to ADC and SCC, but preoperative puncture biopsy is not routinely performed for NSCLC. As such, it was difficult to obtain the pathological type of patients. The application of this model to other pathological types of lung cancer may cause bias in the calculation results.

In conclusion, this study provides a noninvasive prediction tool that combines PET and CT radiomic features and tumor location information. The radiomics nomogram demonstrated good accuracy for identifying occult lymph nodes in NSCLC.

## Data availability statement

The raw data supporting the conclusions of this article will be made available by the authors, without undue reservation.

## Ethics statement

The studies involving human participants were reviewed and approved by Medical Ethics Committee of the Shengjing Hospital,China Medical University. The patients/participants provided their written informed consent to participate in this study. Written informed consent was obtained from the individual(s) for the publication of any potentially identifiable images or data included in this article.

## Author contributions

Conceptualization: JQ and JX. Methodology: JQ, JX, and XZ. Validation: JQ, JX, and XZ. Data curation: JQ, PW, and MD. Formal analysis and investigation: JQ and JX. Software: JQ, XZ, MD, and PW. Writing - original draft: JQ. Writing - review and editing: JX and XZ. Visualization: JQ, MD, and PW. Supervision: JX. Project administration: JX. All authors contributed to the article and approved the submitted version.

## Acknowledgments

Thanks to Yanmei Wang of GE Healthcare for her advice on statistical analysis, Thanks to the scientists of editage for editing our manuscript.

## Conflict of interest

The authors declare that the research was conducted in the absence of any commercial or financial relationships that could be construed as a potential conflict of interest.

## Publisher’s note

All claims expressed in this article are solely those of the authors and do not necessarily represent those of their affiliated organizations, or those of the publisher, the editors and the reviewers. Any product that may be evaluated in this article, or claim that may be made by its manufacturer, is not guaranteed or endorsed by the publisher.
