# Supplementary material for: 18F-FDG PET/CT radiomics nomogram for predicting occult lymph node metastasis of non-small cell lung cancer
Source: Front Oncol. 2022 Sep 28;12:974934. doi: 10.3389/fonc.2022.974934 (PMC9554943; doi:10.3389/fonc.2022.974934)
Supplement: Supplementary file 1 [file DataSheet_1.docx]

**python**

"Ttest_ind", "Levene", "lassocvt" and "extratreesclassifier" in scikit learn are used to screen radiomics characteristics, and "Matplotlib. Pyplot" is used to draw lasso diagram.

**R**

The "RLM" package is used for multivariate logistic regression analysis. The "RMS" package is used to draw radiomics nomograms and calibration curves. "Ggplot2" and "proc" packages are used to draw ROC curves and measure AUC. The "dca" package(Official website of Memorial Sloan Kettering Cancer Center) is used to perform DCA curves. "Ggsci" "hrbrthemes" package to draw violin diagram. The "epiDisplay" package is used to display the OR value and 95% confidence interval of each variable of the logical regression.
